# Genome-wide association and linkage analysis of quantitative traits: comparison of likelihood-ratio test and conditional score statistic

**DOI:** 10.1186/1753-6561-3-s7-s100

**Published:** 2009-12-15

**Authors:** Audrey E Hendricks, Yanyan Zhu, Josée Dupuis

**Affiliations:** 1 Department of Biostatistics, Boston University School of Public Health, 715 Albany Street, Talbot Building, Boston, Massachusetts 02118 USA

## Abstract

Over the past decade, genetic analysis has shifted from linkage studies, which identify broad regions containing putative trait loci, to genome-wide association studies, which detect the association of a marker with a specific phenotype. Because linkage and association analysis provide complementary information, developing a method to combine these analyses may increase the power to detect a true association. In this paper we compare a linkage score and association score test as well as a newly proposed combination of these two scores with traditional linkage and association methods.

## Background

Improvement in genotyping technologies has led to great advances in the quest to map genes influencing complex traits. In the late 1980s came linkage studies in family samples that identified broad regions containing putative trait loci. Recently, dense single-nucleotide polymorphism (SNP) chip technology has resulted in genome-wide association analysis, where the genome is queried for association with a specific phenotype. The high number of SNPs run (from 300,000 to >1,000,000) enables relatively thorough coverage of the genome, but also greatly increases the chance of false-positive results. A low *p*-value in the range of 10^-8 ^is often used to declare genome-wide significance and finding small to moderate associations remains difficult. One advantage of association analysis is it can be carried out in samples of unrelated individuals, which may be easier to recruit. On the other hand, family samples provide extra information about segregation of the phenotype, and both linkage and association analysis may be performed when genotype and phenotype data are available on family members.

Variance-component analysis [1] is a commonly used approach for performing linkage analysis of quantitative traits. Its great flexibility to accommodate extended pedigrees is offset by increased type I error of the likelihood-ratio test (LRT) when the trait is not normally distributed [2]. An alternate approach consists of using the efficient score statistic for linkage analysis standardized by its variance-computed conditional on the observed phenotype [3], which renders it robust to departure from multivariate normality.

Population-based methods can be applied to family samples provided that familial correlation is accommodated and there is no population stratification (or analyses are performed after adjustment for population stratification). Linear mixed-effect models are particularly well suited for association analysis of quantitative traits in family samples. The genotype effect and additional covariates are modeled as fixed effects while familial correlation is accommodated with a random effect with the covariance structure depending on the degree of relatedness between individuals. LRTs may be applied to determine the genotype-phenotype association. Alternatively, an efficient score statistic for association analysis with the variance computed conditional on the observed phenotype may offer a robust option against departure from the normality assumption [4].

The objectives of this paper are two-fold: first, we compare the LRT with conditional score statistics for both linkage and association analyses using the Genetic Analysis Workshop 16 simulated Framingham Heart Study dataset (Problem 3). Second, we explore whether a combination of the linkage and association score improves power to detect associated SNPs.

## Methods

### Framingham Heart Study sample

The subset of simulated Framingham Heart Study samples analyzed in our report consists of 736 families and 381 unrelated individuals. The families range in size from 2 to 291 participants with available genotypes and phenotypes, for a total of 6372 individuals.

Initial data cleaning was performed using the software PLINK [5,6] based on call rate and sex. We removed subjects with a homozygosity rate on the X chromosome between 20% and 80% indicating ambiguous genetic sex, subjects with a conflicting reported and genetic sex, or subjects with a call rate <97%. This resulted in the exclusion of 104 participants.

Lipid-related phenotypes were available at three exams for each participant. To evaluate methods robust to departure from normality, we concentrated on low-density lipoprotein (LDL) and triglyceride levels (TG) levels because of their skewed distributions. We analyzed each trait measured at the first exam, but also averaged each trait over the three exams.

### Chromosome selection

Chromosome 11 was chosen because a group of 39 polygenes influencing high-density lipoprotein (HDL) clustered in the range of 110 Mb to 134 Mb and because of the presence of two major genes, one influencing LDL and the other influencing TG. Chromosome 22 was selected for ease of computation and because it harbors a major gene explaining 1% of the LDL variance.

### Linkage analysis: marker selection

For linkage analysis, we selected markers with low linkage disequilibrium, which may bias identity-by-descent (IBD) estimates when parental information is unavailable [7]. We used PLINK [5,6] to select markers on chromosomes 11 and 22 with call rate ≥ 98%, minor allele frequency of ≥ 35%, a Hardy-Weinberg equilibrium *p*-value ≥ 0.05, and a *r*^2 ^< 0.04. Mendelian transmission errors were detected and corrected using the program PEDCHECK [8].

### Linkage analysis: variance-component model

The basic variance-component model assumes that the vector of phenotype in the *k*^th ^family, *Y*, has a multivariate normal distribution with mean *E*(*Y*|*X*) = *μ*_*X *_= *μ *+ *βX *and covariance matrix: , where X is the covariate of interest;  is the quantitative trait locus, and  and  are the polygenic and residuals variance components, respectively; *π*_*tij *_is the proportion of alleles shared IBD by relatives *i *and *j *at genome location *t*; and *ϕ*_*ij *_is the kinship coefficient. The covariance matrix unconditional on the IBD (Σ) is obtained by setting *π*_*tij *_= *ϕ*_*ij *_in the expression for Σ_*π*_.

The log likelihood for a QTL at *t *is , where the sum is taken over all *N *pedigrees. To test the null hypothesis of no linkage (H_0_:  = 0), one can use the following LRT: . The typical logarithm of odds (LOD) score is computed by dividing this LRT statistic by twice the natural logarithm of 10. The same hypothesis can be tested using the efficient score statistic, which is obtained by taking the first derivative of the log likelihood ratio with respect to , evaluated at  = 0: , where *A*_*π *_is the matrix of centered IBD and *W *= (Σ^-1^(*Y *- *μ*_*X*_)(*Y*- *μ*_*X*_)'-*I*)Σ^-1 ^with elements *w*_*ij*_. The squared of the efficient score is standardized by an estimate of the variance conditional on the observed phenotypes to make it robust to violation of the normality assumption [3,8].

### Association analysis

The linear mixed-effects model to test for association analysis is very similar to the variance-component model, with the exception that the genotype effect is included in the mean rather than in the covariance matrix: *E*(*Y*| *g*, *X*) = *μ *+ *βX *+ *γg*, where *g *is the coded genotypes. The covariance unconditional on the IBD proportions, Σ, is typically used, although one could easily substitute Σ_*π *_at the expense of added computation complexity. The null hypothesis of no association *H*_0_: *γ *= 0 is typically tested using a LRT. Alternatively, the efficient score test may be constructed to test for association. The efficient score statistic for association reduces to , and the estimate of variance, conditional on the observed phenotypes, is , where Φ is the matrix of kinship coefficients. We refer to this statistic as the "conditional score" for association [9].

### Combined linkage and association score

The conditional association score and our implementation of the linkage score are uncorrelated under the null hypothesis of no linkage and no association [10]. Therefore, we summed the chi-square form of these two statistics to produce a combined linkage and association statistic. The asymptotic null distribution of the combined statistic is an equally weighted mixture of chi-square with 1 and 2 degrees of freedom (df) for the additive genetic model and a chi-square mixture with 2 and 3 df for the general 2-df genetic model.

### Evaluation of methods

We computed the LRT and association-conditional score statistics using an additive genetic model and a general 2-df genetic model for each SNP. For all models we adjusted for sex and age, or average age in the case of averaged phenotypes. We compared the association statistics in terms of type I error and power. To determine type I error, we selected SNPs with a call rate above 95%, a HWE *p*-value above 10^-6^, and a low *r*^2 ^(< 0.01) with all polygenes and major genes on chromosomes 11 and 22. We calculated power as the proportion of significant major gene association detected over all 200 phenotype replicates.

We computed multipoint IBD probabilities using the software LOKI [11], making full use of the entire pedigree and all available genotypes. We used the software R [12] to implement the score statistics and the KINSHIP [13] package in R to compute the LRT statistic.

## Results

**Figure 1:**
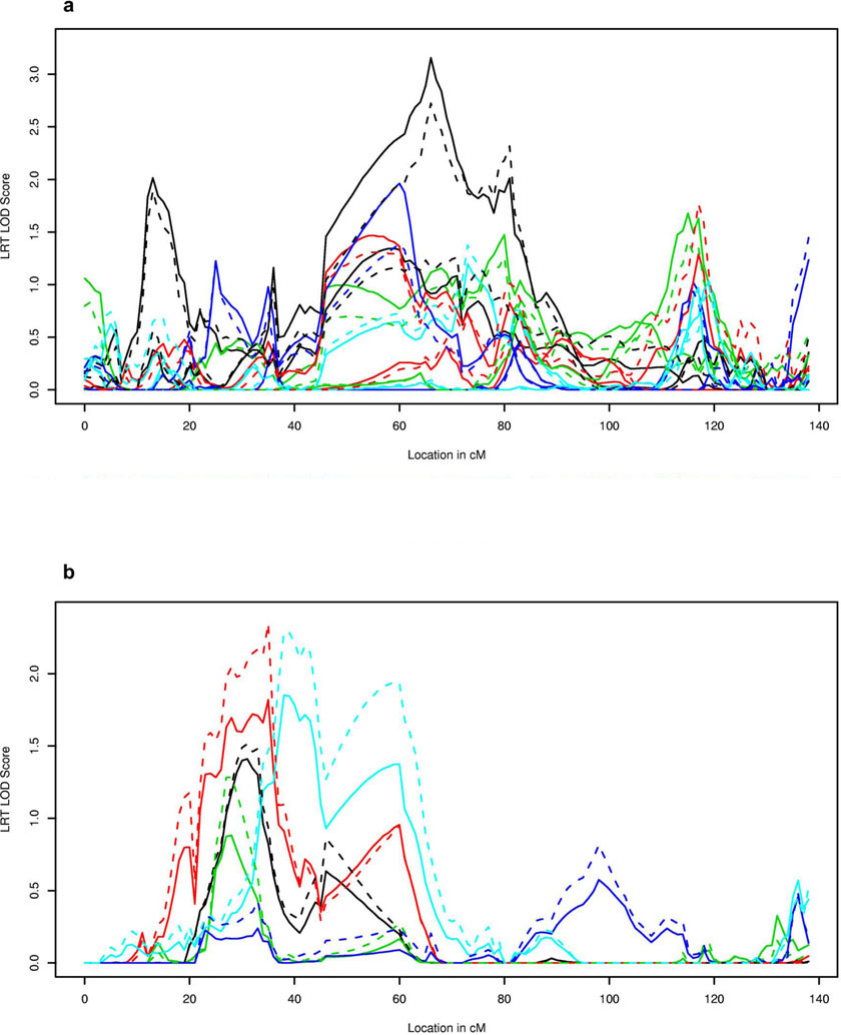
**LDL Visit 1: linkage statistics (a) and TG average: linkage statistics (b)**. LOD score on chromosome 11: linkage score statistic (solid lines) and LRT (dashed lines). Simulation 1, black; 2, red; 3, green; 4, dark blue; 5, light blue.

Figure 1 presents the linkage signals produced for the LRT and linkage-conditional score for Simulations 1-5. The maximum LOD score of 3.2 for the score statistic (LRT LOD = 2.7) was attained for LDL at Visit 1, Simulation 1 (Figure 1a). The LOD score curve for the average LDL over the three visits has a similar shape, with reduced evidence for linkage in Simulation 1 (maximum linkage score = 1.6 and maximum LRT LOD= 2.1; results not shown). The maximum LOD score for TG average was 1.9 in Simulation 5 (maximum LRT LOD = 2.3; Figure 1b), but was much reduced for TG at Exam 1 (maximum LOD score = 0.6; maximum LRT LOD = 0.8).

As seen in Table 1, the type I error for association analysis assuming the additive model is comparable for the association-conditional score and the LRT. There is a negligible increase in type I error for the general 2-df LRT, while the increase is moderate for the conditional score. Similar trends for type I error are seen for average LDL and TG at Visit 1 (data not shown).

**Table 1:** Type I error

	LDL---Visit 1				TG---Average			
		
	0.050	0.010	0.0010	0.00010	0.050	0.010	0.0010	0.00010
LRT (additive)								
MAF>1%	0.048	0.012	0.0019	0.00010	0.051	0.012	0.0013	0.00015
MAF>10%	0.047	0.012	0.0018	0.00013	0.050	0.012	0.0010	0.00013
LRT (general 2 df)								
MAF>1%	0.053	0.012	0.0012	0.00031	0.055	0.015	0.0039	0.00191
MAF>10%	0.050	0.011	0.0011	0.00025	0.050	0.012	0.0009	0.00013
Association conditional score (additive)								
MAF>1%	0.045	0.010	0.0013	0.00005	0.050	0.010	0.0012	0.00010
MAF>10%	0.044	0.009	0.0014	0.00006	0.048	0.010	0.0009	0.00013
Association conditional score (general 2 df)								
MAF>1%	0.065	0.016	0.0029	0.00118	0.073	0.023	0.0060	0.00282
MAF>10%	0.061	0.014	0.0013	0.00019	0.067	0.017	0.0021	0.00031

**Figure 2:**
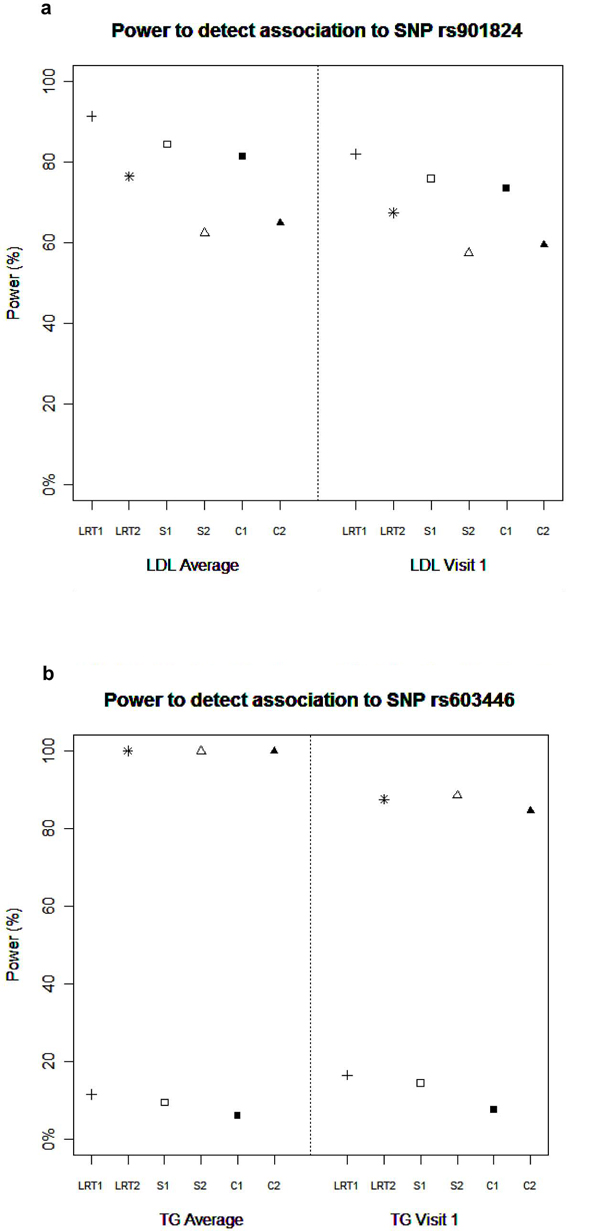
**Power to detect association to SNP rs901824 (a) and SNP rs603446 (b)**. Power for association statistics on chromosome 11. Power was calculated at *α *= 0.05 significance level. LRT1, likelihood-ratio test additive model; LRT2, likelihood-ratio test general 2-df model; S1, conditional score additive model; S2, conditional score general 2-df model; C1, combined conditional score additive model; C2, combined conditional score general 2-df model.

Power to detected major SNPs at *α *= 5% is presented in Figure 2, and is similar between the LRT, association-conditional score, and combined conditional score when looking across the same genetic model. Assuming an additive genetic model, all statistics have very low power to detect the association between TG levels and rs603446, an over-dominant SNP for which carriers of the heterozygote genotypes have higher simulated TG levels. All of the general 2-df model statistics have much higher power at *α *= 5% to detect this association as shown in Figure 2; there was minimal power (<10%) at a more stringent significance level of *α *= 10^-8 ^(data not shown). Power to detect association between rs901824 and LDL ranged between 60% and 80% at *α *= 5%, but power was 0 for all statistics at *α *= 10^-8 ^(results not shown). All methods achieved 100% power to detect SNP rs2294207, an additively acting SNP on chromosome 22 explaining 1% of the LDL variation with either LDL phenotypes (data not shown).

## Discussion

Despite selecting phenotypes that violated the normality assumption, type I error rates for the additive association-conditional score and LRT statistics were not inflated, but slight type I error inflation was observed for the 2-df LRT statistic and moderate inflation for the 2-df association-conditional score. The type I error rate inflation for the 2-df statistics is most likely due to a violation of asymptotic assumptions caused by SNPs with low cell counts. Note that filtering by MAF less than 10% lowers the false-positive rate for both 2-df statistics.

In our analyses, all additive statistics failed to detect the association between TG levels and rs603446, a SNP with a non-additive effect. This result suggests, as do Lettre et al. [14], that the additive model has low power to detect associations for genetic models in which the association is limited to only one genetic group, such as recessive or over-dominant genetic models. Although power is lost when the general 2-df model is used to detect additive SNPs, the reduction is much smaller than the power reduction when using the additive model to detect the over-dominant SNP. Because the genetic model of a SNP is always unknown a priori, the general 2-df model seems to be the better model to detect an association.

Including the information from the linkage analysis using the combined conditional score did not consistently increase the power to detect an associated SNP. However, despite the lack of a large linkage signal and even though the combined conditional score statistic uses an extra degree of freedom, the power remained fairly constant when using the combined conditional score compared with the LRT and the association-only conditional score. The minimum LOD score needed at *α *= 0.05 to accommodate the extra degree of freedom used by the combined conditional score is 0.467 for the additive model and 0.396 for the general 2-df model. The percent of iterations that reached the minimum threshold ranged from 4.0% for the additive model for visit 1 TG levels at SNP rs603446 to 31.5% for the general 2-df model for average LDL at SNP rs901824. This suggests that a larger linkage signal or a more efficient combination of the conditional association and linkage scores may ultimately increase power.

A notable difference between the LRT and the conditional score for association was computation time. Performing the analysis for a total of 35,979 SNPs on chromosomes 11 and 22 using a single processor would take more than 16 hours using the LRT and less than an hour using the association-conditional score. The computation times for the linkage analysis were more comparable. Once the IBD was computed, the LRT using SOLAR took about 45 minutes to scan chromosome 11, while the linkage-conditional score took about 3.25 hours. Despite the longer linkage computation time, the combined conditional score was computed much faster than the LRT.

Although more research is needed, conditional score analysis provides an interesting possibility of a gain in power by combining linkage information using the combined conditional score. Regardless, the conditional score for association provides a fast and comparable alternative to LRTs for analysis of family data.

## List of abbreviations used

HDL: High-density lipoprotein; IBD: Identity by descent; LDL: Low-density lipoprotein; LOD: Logarithm of the odds; LRT: Likelihood ratio test; SNP: Single-nucleotide polymorphism; TG: Triglyceride

## Competing interests

The authors declare that they have no competing interests.

## Authors' contributions

AEH performed the initial data cleaning, carried out the LRT association analysis, and wrote and revised the manuscript. YZ carried out the variance-component linkage analysis and revised the manuscript. JD conceived the study, implemented the conditional score analysis, and drafted and revised the manuscript. All authors read and approved the final manuscript.
